# Evaluation of the Learning Curve Threshold in Robot-Assisted Lung Cancer Surgery: A Nationwide Population-Based Study

**DOI:** 10.3390/cancers16244221

**Published:** 2024-12-18

**Authors:** Pierre-Benoit Pages, Jonathan Cottenet, Leslie Madelaine, Florian Dhérissard, Halim Abou-Hanna, Alain Bernard, Catherine Quantin

**Affiliations:** 1 Department of Thoracic and Cardiovascular Surgery, CHU Dijon Bourgogne, 21000 Dijon, France; pierrebenoit.pages@chu-dijon.fr (P.-B.P.); leslie.madelaine@chu-dijon.fr (L.M.); florian.dherissard@chu-dijon.fr (F.D.); halim.abouhanna@chu-dijon.fr (H.A.-H.); alain.bernard@chu-dijon.fr (A.B.); 2 Service de Biostatistiques et d’Information Médicale (DIM), CHU Dijon Bourgogne, 21000 Dijon, France; jonathan.cottenet@chu-dijon.fr; 3 INSERM, Université de Bourgogne, CIC 1432, Module Épidémiologie Clinique, 21000 Dijon, France; 4 Inserm, Centre de recherche en épidémiologie et santé des populations, Université de Versailles Saint-Quentin-en-Yvelines, Université Paris-Saclay, 94807 Villejuif, France

**Keywords:** lung cancer, robot-assisted surgery, learning curve, postoperative complications, hospital database

## Abstract

Background: Recent publications suggest that the threshold for validation of the learning curve is 25 procedures. The aim of this study was to evaluate this threshold using another rarely used method, based on a composite quality indicator. Methods: We included all patients from the French medico-administrative database receiving robot-assisted surgery for lung cancer, with a focus on hospitals performing at least 25 procedures over the period 2019–2022. For postoperative complication analysis, we used the Clavien–Dindo classification. We used the sequential probability ratio test to estimate the number of procedures at which a hospital achieved its learning curve. Results: In France, the number of robotic-assisted procedures has risen steadily in the past few years: 195 in 2019 and 1567 in 2022 (overall, 3706 Robot-Assisted surgeries). The total number of patients with Clavien–Dindo classification > II was 833 (24.7%). Among the 28 hospitals performing at least 25 procedures, eight achieved their learning curve with thresholds ranging from 94 to 174 procedures, and the median was 110. Severe complications such as acute respiratory distress syndrome, respiratory failure, heart failure, acute ischemia of the lower limbs, or pulmonary embolism were significantly more frequent in the group of hospitals that did not validate the learning curve threshold. Conclusions: This study suggests that the threshold of 25 procedures may not be sufficient to validate the robot-assisted surgery learning curve in lung cancer surgery. To significantly reduce postoperative complications, a hospital would need to perform 94 to 174 procedures to guarantee patient safety.

## Simple Summary

Recent publications suggest that the threshold for validation of the learning curve is 25 procedures. The aim of this study was to evaluate this threshold using another infrequently used method based on a composite quality indicator. From the French medico-administrative database, we used the sequential probability ratio test to estimate the number of robot-assisted surgeries for lung cancer at which a hospital achieved its learning curve, using the Clavien–Dindo classification as the quality indicator. Overall, 28 hospitals performed 3707 Robot-Assisted surgeries for lung cancer between 2019 and 2022, with a total number of patients with Clavien–Dindo classification > II of 833 (24.7%). We found that the threshold of 25 procedures may not be sufficient to validate the robot-assisted surgery learning curve in lung cancer surgery. In order to guarantee an acceptable complication rate, a hospital would need to perform 94 to 174 procedures.

## 1. Introduction

Robot-assisted (RA) surgery is gaining ground in the treatment of operable lung cancer (LC) [1,2,3,4]. For early-stage LC, recommendations call for minimally invasive approaches, which includes RA surgery. In addition to understanding the clinical benefits of these innovative technologies, we believe it is important to assess their potential risks and complications.

The diffusion of any new surgical method requires a learning curve assessment to estimate a threshold at which surgeons should be able to master the technology. In a recent systematic literature reviews estimating the learning curve threshold [5], the studies have mostly focused on procedure duration, conversions, and intraoperative complications to determine the learning curve threshold [6,7]. At this stage in the dissemination of RA thoracic surgery, we felt it was important to analyze the results in terms of postoperative quality indicators, rather than focusing solely on intraoperative criteria. This recent meta-analysis estimated that an average of 25 procedures were needed to validate the learning curve [5]. However, this threshold depends on the outcomes and the methods used (Table S1).

Based on this literature data, we aim to verify if the threshold of a minimum of 25 procedures guarantees that patients receive quality care with an acceptable rate of postoperative complications. As an indicator, we used postoperative complications and/or mortality occurring in the 30 days following the procedure. To assess postoperative complication, we used the Clavien–Dindo classification, which is widely accepted in the literature [8].

The aim of this study was to evaluate the learning curve threshold of hospitals that performed at least 25 lung cancer resections by RA Thoracic Surgery using the Clavien–Dindo classification as the judgment criterion.

## 2. Materials and Methods

The French national hospital database (PMSI) is a system inspired by the US Medicare system. The PMSI provides detailed medical information on all admissions to public and private hospitals in France. The data include discharge diagnoses according to the tenth edition of the International Classification of Diseases (ICD-10) [9,10] and medical procedures coded according to the Common Classification of Medical Procedures (CCAM).

We included all patients with a pulmonary resection between 2019 and 2022. These patients were identified in the PMSI through a principal discharge diagnosis of LC (ICD-10 code C34) and an intervention for LC of RA thoracic surgery (CCAM code GFFA009-40) during the same hospital stay [11]. For all patients, an LC diagnosis was established by pathological analysis according to the World Health Organization’s 2004 classification of lung tumors [9].

### 2.1. Patient Characteristics

At baseline, we identified patient age and sex and surgery-related variables including the surgical approach and the type of resection (limited resection, lobectomy). We also included the following comorbidities: pulmonary disease (chronic bronchitis and emphysema), heart disease (coronary artery disease, cardiac arrhythmia, congestive heart failure, valvular heart disease, pulmonary artery hypertension, or pulmonary embolism), peripheral vascular disease, liver disease, cerebrovascular events, neurological diseases (hemiplegia or paraplegia), renal disease, hematologic disease (leukemia or lymphoma), metabolic disease (including obesity), anemia, other therapies (preoperative chemotherapy including neoadjuvant therapies or steroids), and infectious disease. Finally, we calculated a modified Charlson Comorbidity Index (CCI) as a marker of comorbidity [12]. This score assesses the comorbidity level by taking the severity of 19 pre-defined comorbid conditions (myocardial infarction, congestive heart failure, peripheral vascular disease, cerebrovascular disease, dementia, chronic pulmonary disease, connective tissue disease, ulcer disease, mild liver disease, diabetes, hemiplegia, moderate or several renal disease, diabetes with end organ damage, any tumor, leukemia, lymphoma, moderate or severe liver disease, metastatic solid tumor, and AIDS) into account. Each condition is assigned a weight from 1 to 6 (6 for the more severe comorbidities), which provides a weighted score of a patient’s comorbidities that can be used to predict short-term and long-term mortality [12].

### 2.2. Hospital Characteristics

French hospitals are classified as non-teaching public, teaching public, private non-profit, or private for profit. For each hospital, we also calculated the total hospital volume, defined as the number of thoracic procedures performed for LC and the total robot-assisted procedure during the same period. It should be noted that, in France, a single team within the hospital performs this type of surgery.

### 2.3. Ethics

The French national hospital data are based on pseudonymized data, i.e., they do not contain any identifying data. Patient consent was therefore not required for this study seeing as patient-identifying information was not used. The patient’s identity is pseudonymized in the PMSI, which allows data from the same patient to be linked without revealing their identity. The study was conducted according to the guidelines of the Declaration of Helsinki and approved by the National Committee for data protection: declaration of conformity to the methodology of reference [5] obtained on 7 August 2018 under the number 2204633 v0.

We are not allowed to transmit these data. PMSI data are available for researchers who meet the criteria for access to these French confidential data (this access is submitted for the approval of the National Committee for data protection) from the national agency for the management of hospitalization (ATIH—Agence technique de l’information sur l’hospitalisation).

### 2.4. Outcome Measurements

Thirty-day mortality was defined as deaths occurring during the surgical stay or during a subsequent hospital stay within 30 days of admission for the surgical stay.

We also considered the presence of one or more of the following other postoperative conditions: pain, wound complication, tracheostomy, reintubation, adult respiratory distress syndrome, bronchopleural fistula, empyema, respiratory failure, arrythmia, malnutrition, phlebitis, pleural effusion, pulmonary embolus, pneumonia, hemorrhage, myocardial infarction, stroke, lower limb ischemia, sepsis, and heart failure.

For the analysis, we used the Clavien–Dindo classification for 30-day mortality and other postoperative complications [8]. The Clavien–Dindo classification was transformed into a binary variable. The new variable was equal to 1 if the Clavien–Dindo classification was > II, which included 30-day mortality (grade V) and other postoperative complications (grades III–IV) (Table S2).

### 2.5. Statistical Analysis

Descriptive data were expressed as *n* (%) for qualitative variables and as mean ± standard deviation for continuous variables. Means were compared using a parametric test (ANOVA). Categorical variables were compared using Χ2 test. We first described the characteristics of patients among all hospitals performing RA surgeries and then focused on patients operated in hospitals performing at least 25 procedures over the period 2019–2022. To estimate the predicted risk of failure (Clavien–Dindo classification > II), we used a logistic regression model. The discriminative ability of the model was expressed by the area under the receiver operating characteristic curve (AUC). The reliability of the model was assessed using the Hosmer–Lemeshow goodness-of-fit test [13].

We used the sequential probability ratio test (SPRT) and its modification—the risk-adjusted sequential probability ratio test (RA-SPRT) [14]. In medical practice, SPRT is one of the statistical tests used to monitor the safety of medical interventions [14,15,16]. The SPRT has been used for LC assessment in several studies [14,15,16], where boundary lines were constructed to detect an increase in the failure rate equivalent to a 50% increase in risk (odds ratio, 1.5). The detailed methodology to build the graph is available in the appendix 1 described in Rogers et al. [14]. False-positive (α) and false-negative (β) error rates were respectively 5% and 20% to build upper (h1) and lower (h0) boundary lines. When the lower boundary line (h0) was crossed by the cumulative outcome curve (Ti), the null hypothesis was accepted, which indicated that an acceptable performance was achieved. To estimate the number of procedures from which a hospital achieved its learning curve, the SPRT curve had to cross the lower boundary line (h0), calculated using the formula described by Rogers et al. [14]. In order to check that the threshold found by this method is robust, we also performed a spline analysis using mixed-effects logistic regression. We also estimated the threshold on lobectomy only as a sensitivity analysis.

Calculations were performed with STATA V.18 statistical software (StataCorp, College Station, TX, USA) and Excel software (version 16.0).

## 3. Results

Between 2005 and 2020, 3706 patients were operated on for LC in France using the RA approach. The number of RA procedures rose considerably in the last few years, from 195 procedures in 2019 to 1567 in 2022. Patient characteristics over the last 4 years are shown in Table 1. Most comorbidities remained stable over time, except for the ‘other disease and other treatment’ variable, which varied significantly. The CCI score varied significantly (*p* = 0.028) over time, particularly for score 0 and score ≥ 3 (Table 1). The type of lung resection did not vary over the 4 years (Table 1). Finally, the variation in Clavien–Dindo classification was at the limit of significance (*p* = 0.048), with the greatest fluctuations in group I, group II, and group IVa (Table 1). In 2022, 30-day mortality (group V) dropped to a rate of 0.6% (Table 1).

### 3.1. Hospital Characteristics

The number of hospitals performing RA procedures has increased steadily from 2019 to 2022, rising from 29 in 2019 to 64 in 2022 (Table 2). Hospitals belonging to teaching hospitals performed 46.8% of pulmonary resections by RA surgery in 2022 (Table 2). We found that the hospitals using RA surgery are high-volume hospitals (Table 2). From 2019 to 2022, 28 centers performed at least 25 RA procedures over the period (Table 2). These hospitals are included in the estimation of the learning curve threshold using the Clavien–Dindo classification.

### 3.2. Predicted Risk Model

The total number of patients with Clavien–Dindo classification > II was 833 (24.7%). The logistic regression model is reported in Table S3. In the model, we included 17 variables (Table S3). This model had good performance with AUC ROC of 0.887. The Hosmer–Lemeshow goodness-of-fit test was non-significant for this model (*p* = 0.275).

### 3.3. Learning Curve Threshold

**Figure 1:**
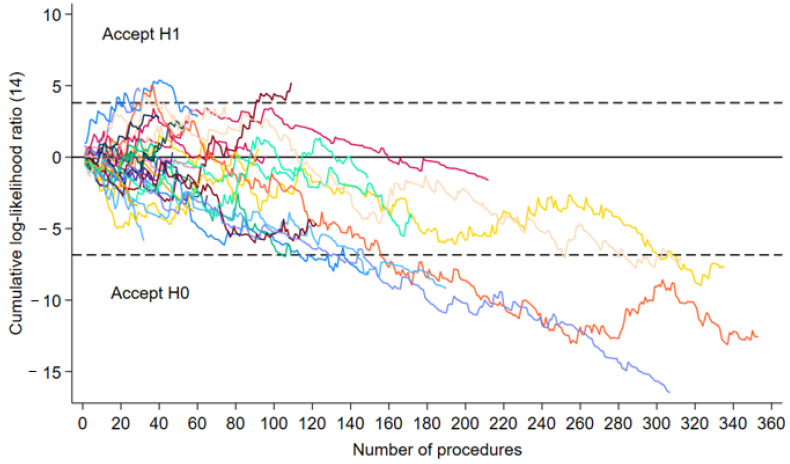
Risk-adjusted sequential probability ratio test chart for 28 hospitals that performed at least 25 robot-assisted thoracic surgeries during the 2019 to 2022 period. The lower boundary line (h0) had a value of −6.838 (α = 0.05, β = 0.2, odds ratio = 1.5). Each of the 28 hospitals is represented by a colored line, making it possible to estimate the number of procedures at which a hospital has reached its learning curve.

The sequential probability ratio test (SPRT) graphs of the 28 hospitals that performed at least 25 procedures are shown in Figure 1. To estimate the number of procedures from which a hospital achieved its learning curve, the SPRT curve had to cross the lower boundary line (h0), which had a value of −6.838 (α = 0.05, β = 0.2, odds ratio = 1.5). Among the 28 hospitals, eight hospitals achieved their learning curve as the graph crossed the lower boundary line. The learning curve threshold for the eight hospitals ranged from 94 to 174 procedures, with a median of 110 procedures. Based on a spline analysis using mixed-effects logistic regression, we found that the result was more or less the same, with a threshold of 110 procedures (Figure S1). The median number of procedures performed between 2019 and 2022 by the eight hospitals that reached the learning curve threshold was 248, with a minimum of 109 and a maximum of 353.

**Figure 2:**
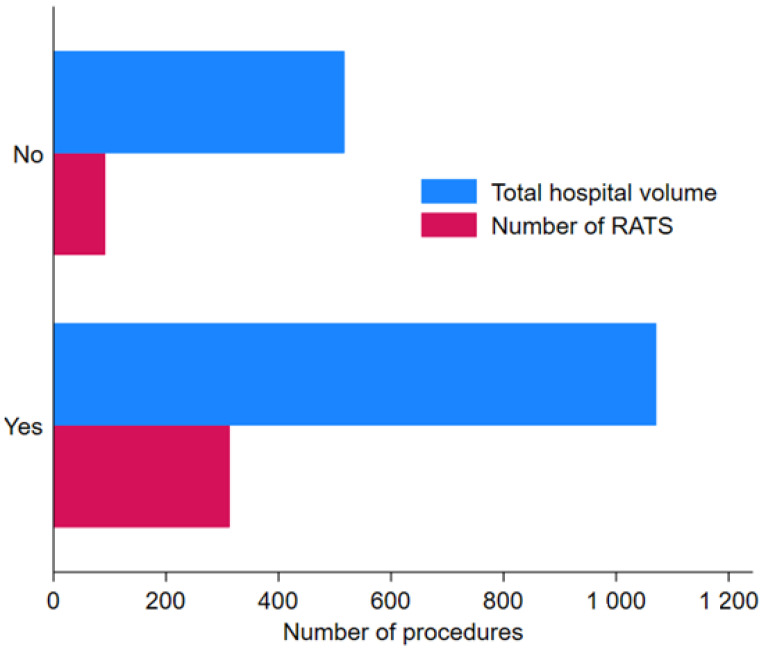
Comparison of total hospital volume and number of robot-assisted thoracic surgeries in hospitals that reached the learning curve (*n* = 8) and hospitals that failed to validate the learning curve (*n* = 20).

Figure 2 shows that hospitals that reached their learning curve have a median hospital volume for all types of lung resection for LC (thoracotomy, VATS and RATS) of 1031, compared with 516 for hospitals that have not reached the learning curve threshold. Among the eight hospitals, four were teaching hospitals, one a non-teaching hospital, one a private non-profit hospital and two private for-profit hospitals.

Operative details and postoperative outcomes of both groups are shown in Table 3. The eight hospitals that reached the learning curve threshold performed a total of 1870 RA procedures for LC, compared to 1489 at the 20 hospitals that did not reach the learning curve threshold (Table 3). The rate of hemorrhage complications was 4.6% in the group that did not reach the learning curve, compared to 3.1% in the group that did (*p* = 0.02) (Table 3). Postoperative pleural effusion occurred in 16.1% of patients in a hospital that did not reach the learning curve, compared to 13.1% in a hospital that did (*p* = 0.016) (Table 3). Severe complications such as ARDS, respiratory failure, heart failure, acute ischemia of the lower limbs, and pulmonary embolism were significantly more frequent in the group of hospitals that did not reach the learning curve threshold (Table 3). Postoperative mortality was 1.3% in the group that did not reach the learning curve, compared to 1.1% in the group that did (*p* = 0.685) (Table 3).

## 4. Discussion

Our findings indicate that a hospital performing RA procedures for LC should have performed at least 110 procedures, with a range of 94 to 174, in order to guarantee patients an acceptable complication rate. Most studies that have examined the learning curve in robot-assisted surgery use different outcomes, such as operative duration, conversion to thoracotomy, or intraoperative accidents [17,18,19,20]. These criteria are obviously necessary but may not be sufficient to demonstrate that the technology is safe and provides patients with a high quality of care. The relevance of operating time in assessing quality of care also seems relevant, leading to the question of whether a surgeon who requires a longer operating time may put patients at risk [18,19,20].

The hospitals that reached the learning curve threshold are high-volume hospitals. It is possible that our study evaluates not only the learning curve but also the performance of the centers, which is usually better in hospitals with a high volume of activity [21]. The number of RA procedure for LC increased steadily from 2019 to 2022, and we found that the RA surgery program was offered in high-volume hospitals. This seems to confirm the results we obtained in a previous study [22] before showing a great variability from one region to another in the spread of these minimally invasive technologies. In addition, patients in some regions may not have access to these innovative technologies because the robots are not available in the lower volume centers, preventing surgical teams in these centers from mastering the minimally invasive approach [22].

Here, the rate of postoperative hemorrhagic complications and pleural effusions decreased in hospitals with more experience performing RA procedures. This is consistent with the results by Zhang et al. [23], who reported lower complication rates than in our study. However, this could be explained by the fact that teams included in Zhang et al. were from high-volume centers. The significant reductions in major complications such as ARDS, heart failure, or acute ischemia of the lower limbs may be surprising. We can imagine that the lack of mastery of the technology may have consequences for the postoperative course and a major impact on patients. Finally, few papers reported a high rate of severe postoperative complications. To the best of our knowledge, only two papers have reported lower rates of these complications than in our work [23,24]. The higher frequency of major postoperative complications in our work can be explained by a longer procedure or serious intraoperative accidents such as vessel injury leading to major hemorrhage.

Over the study period, the 30-day mortality rate decreased from a high of 1.9% the year before, falling to 0.6% in 2022. This substantial advance is certainly associated with the use of minimally invasive techniques [21].

The prolonged length of stay did not decrease in hospitals that validated the learning curve. In the literature, lower rates have been reported [17,18,20,23,24]. However, comparisons with other countries are difficult seeing as organization and funding differ. The main issue in France is the organization of patient pathways and, more particularly, the preparation for hospital discharge, in collaboration with out-of-hospital care.

Our study has the usual limitations of medico-administrative studies including the absence of variables such as TNM stage or FEV1. However, recent results have proven the good performance of predictive models developed from medico-administrative data and a clinical database [25]. Another limitation concerns 30-day mortality, which may be considered a less relevant indicator than 90-day mortality. However, 90-day mortality may not reflect the quality of surgery alone or be the best indicator for estimating the learning curve considering that other factors such as adjuvant chemotherapy, radiotherapy or disease progression may cause death during the 90-day period. In addition, the literature on this subject remains inconclusive as there is no information on missing data and the performance of the model is not high [26]. Further studies are thus needed to validate 90-day mortality as an indicator of surgical quality. The lack of intraoperative variables such as the length of stay or conversion, which cannot be collected in a database such as the PMSI, is another limitation of this work. Finally, the evaluation of the learning curve concerned the surgical team and not the surgeon alone as we were only able to evaluate the overall practice of a hospital’s surgical team because the PMSI does not collect information on the practice of individual surgeons. Indeed, it is not possible to identify the different French practitioners who performed surgeries in our national cohort or to ascertain their experience. However, in France, at the current stage of the use of RA surgeries, hospitals have a single team performing this type of surgery, with one to three experienced surgeons able to perform them, most of them having just one.

Finally, this work is innovative in terms of estimating the learning curve for robotic surgery, since the outcome chosen is postoperative complications. This indicator seems to us more appropriate for measuring the quality of the surgical gesture than the measure of operating time as reported in most studies. This work will enable teams new to robotic surgery to use this indicator to validate their learning curve and to be supported by teams who have already validated their learning curve. Indeed, in future, the type of data used in this study could be provided in the form of a dashboard to surgical teams initiating a robotic surgery program, enabling them to follow their learning curve and, if necessary, to implement measures to improve performance.

## 5. Conclusions

The threshold of 25 procedures does not appear sufficient to validate the robot-assisted surgery learning curve in LC surgery. A hospital would need to perform between 94 and 174 procedures to significantly reduce postoperative complications, thereby ensuring patient safety.

## Figures and Tables

**Table 1:** Characteristics of patients who underwent robot-assisted thoracic surgery from 2019 to 2022, whatever the volume of activity.

	2019	2020	2021	2022	*p*-Value
	*n* = 195	*n* = 747	*n* = 1197	*n* = 1567	
Age (years) ^$^	67.24 (9)	66.7 (9.3)	67.2 (8.8)	66.96 (8.8)	0.769
Sex					
Male	117 (60.0%)	412 (55.2%)	655 (54.7%)	864 (55.1%)	0.588
Female	78 (40.0%)	335 (44.8%)	542 (45.3%)	703 (44.9%)	
Pulmonary disease					
No	146 (74.9%)	545 (73.0%)	863 (72.1%)	1136 (72.5%)	0.870
Yes	49 (25.1%)	202 (27.0%)	334 (27.9%)	431 (27.5%)	
Heart disease					
No	162 (83.1%)	634 (84.9%)	1031 (86.1%)	1349 (86.1%)	0.596
Yes	33 (16.9%)	113 (15.1%)	166 (13.9%)	218 (13.9%)	
Peripheral vascular disease					
No	183 (93.8%)	681 (91.2%)	1097 (91.6%)	1424 (90.9%)	0.542
Yes	12 (6.2%)	66 (8.8%)	100 (8.4%)	143 (9.1%)	
Neurological disease					
No	189 (96.9%)	711 (95.2%)	1146 (95.7%)	1499 (95.7%)	0.758
Yes	6 (3.1%)	36 (4.8%)	51 (4.3%)	68 (4.3%)	
Cirrhosis					
No	195 (100.0%)	740 (99.1%)	1181 (98.7%)	1559 (99.5%)	0.061
Yes	0 (0.0%)	7 (0.9%)	16 (1.3%)	8 (0.5%)	
Renal disease					
No	189 (96.9%)	712 (95.3%)	1156 (96.6%)	1519 (96.9%)	0.250
Yes	6 (3.1%)	35 (4.7%)	41 (3.4%)	48 (3.1%)	
Metabolic disease					
No	161 (82.6%)	646 (86.5%)	1014 (84.7%)	1342 (85.6%)	0.481
Yes	34 (17.4%)	101 (13.5%)	183 (15.3%)	225 (14.4%)	
Anemia					
No	163 (83.6%)	649 (86.9%)	1002 (83.7%)	1313 (83.8%)	0.216
Yes	32 (16.4%)	98 (13.1%)	195 (16.3%)	254 (16.2%)	
Infectious disease					
No	194 (99.5%)	747 (100.0%)	1190 (99.4%)	1563 (99.7%)	0.145
Yes	1 (0.5%)	0 (0.0%)	7 (0.6%)	4 (0.3%)	
Hematologic disease					
No	190 (97.4%)	729 (97.6%)	1166 (97.4%)	1536 (98.0%)	0.736
Yes	5 (2.6%)	18 (2.4%)	31 (2.6%)	31 (2.0%)	
Other disease					
No	135 (69.2%)	430 (57.6%)	695 (58.1%)	998 (63.7%)	<0.001
Yes	60 (30.8%)	317 (42.4%)	502 (41.9%)	569 (36.3%)	
Other treatment					
No	182 (93.3%)	645 (86.3%)	1046 (87.4%)	1439 (91.8%)	<0.001
Yes	13 (6.7%)	102 (13.7%)	151 (12.6%)	128 (8.2%)	
Charlson score					
0	82 (42.1%)	283 (37.9%)	445 (37.2%)	614 (39.2%)	0.028
1	26 (13.3%)	69 (9.2%)	130 (10.9%)	195 (12.4%)	
2	24 (12.3%)	88 (11.8%)	120 (10.0%)	190 (12.1%)	
≥3	63 (32.3%)	307 (41.1%)	502 (41.9%)	568 (36.2%)	
Pulmonary resection					
limited	8 (4.1%)	21 (2.8%)	37 (3.1%)	36 (2.3%)	0.382
lobectomy	187 (95.9%)	726 (97.2%)	1160 (96.9%)	1531 (97.7%)	
Clavien–Dindo					
None	118 (60.5%)	426 (57.0%)	698 (58.3%)	938 (59.9%)	0.048
Group ≤ II	25 (12.8%)	138 (15.0%)	196 (16.3%)	241 (15.4%)	
Group IIIa	21 (10.8%)	55 (7.4%)	108 (9.0%)	132 (8.4%)	
Group IIIb	6 (3.1%)	21 (2.8%)	36 (3.0%)	57 (3.6%)	
Group IVa	2 (1.0%)	23 (3.1%)	27 (2.3%)	40 (2.6%)	
Group IVb	20 (10.3%)	73 (9.8%)	109 (9.1%)	150 (9.6%)	
Group V	3 (1.5%)	11 (1.5%)	23 (1.9%)	9 (0.6%)	

^$^ mean (standard deviation). Footnote: limited resection included wedge or segmentectomy, pulmonary disease (chronic bronchitis or emphysema), heart disease (coronary artery disease, cardiac arrhythmia, congestive heart failure, valvular heart disease, pulmonary artery hypertension, or pulmonary embolism), peripheral vascular disease, liver disease, cerebrovascular events, neurological diseases (hemiplegia or paraplegia), renal disease, hematologic disease (leukemia or lymphoma), metabolic disease (including obesity), anemia, other therapies (preoperative chemotherapy including neoadjuvant therapies or steroids), and infectious disease.

**Table 2:** Characteristics of hospitals performing robot-assisted (RA) thoracic surgery from 2019 to 2022, whatever the volume of activity.

Year					
	2019	2020	2021	2022	*p*-Value
No. of patients	195	747	1197	1567	
No. of hospitals	29	50	43	64	
No. of hospitals > 25 procedure	20	25	25	28	
Type of hospital					
Non-teaching	37 (19.0%)	114 (15.3%)	128 (10.7%)	161 (10.3%)	<0.001
Teaching	30 (15.4%)	264 (35.3%)	548 (45.8%)	733 (46.8%)	
Private non-profit	53 (27.2%)	127 (17.0%)	203 (17.0%)	234 (14.9%)	
Private for profit	75 (38.5%)	242 (32.4%)	318 (26.6%)	439 (28.0%)	
Total hospital volume ^$^	187 (222)	184 (189)	236 (210)	263 (268)	<0.001
Hospital volume RA ^$^	13 (6.3)	44 (29.7)	69 (39.6)	72 (52)	<0.001
Clavien Dindo > II					
Observed ^$$^	0.15 [0–0.41]	0.24 [0.19–0.32]	0.25 [0.19–0.33]	0.27 [0.17–0.37]	
Predicted ^$$^	0.21 [0.12–0.32]	0.22 [0.15–0.32]	0.25 [0.18–0.32]	0.24 [0.18–0.356]	

^$^: Mean (Standard deviation), Frequency (Percent %), ^$$^: Median [1st Quartile–3rd Quartile].

**Table 3:** Procedural and outcome variables of patients in hospitals performing at least 25 robot-assisted thoracic surgeries over the period 2019 to 2022.

Threshold Learning Curve			
	No	Yes	*p*-Value
	*n* = 1489	*n* = 1870	
Pulmonary resection			
limited	39 (2.6%)	43 (2.3%)	0.551
Lobectomy	1450 (97.4%)	1827 (97.7%)	
Clavien–Dindo			
No	847 (56.9%)	1136 (60.7%)	<0.001
≤II	231 (15.5%)	317 (17.0%)	
IIIa	115 (7.7%)	170 (9.1%)	
IIIb	56 (3.8%)	54 (2.9%)	
IVa	45 (3.0%)	41 (2.2%)	
IVb	176 (11.8%)	131 (7.0%)	
V	19 (1.3%)	21 (1.1%)	
Fistula			
No	1475 (99.1%)	1857 (99.3%)	0.429
Yes	14 (0.9%)	13 (0.7%)	
Empyema			
No	1483 (99.6%)	1859 (99.4%)	0.452
Yes	6 (0.4%)	11 (0.6%)	
Pneumonia			
No	1392 (93.5%)	1729 (92.5%)	0.250
Yes	97 (6.5%)	141 (7.5%)	
Hemorrhage			
No	1420 (95.4%)	1812 (96.9%)	0.021
Yes	69 (4.6%)	58 (3.1%)	
Pleural effusion			
No	1250 (83.9%)	1625 (86.9%)	0.016
Yes	239 (16.1%)	245 (13.1%)	
Respiratory failure			
No	1332 (89.5%)	1756 (93.9%)	<0.001
Yes	157 (10.5%)	114 (6.1%)	
ARDS			
No	1426 (95.8%)	1831 (97.9%)	<0.001
Yes	63 (4.2%)	39 (2.1%)	
Arrhythmia			
No	1322 (88.8%)	1654 (88.4%)	0.761
Yes	167 (11.2%)	216 (11.6%)	
Myocardial infarction			
No	1478 (99.3%)	1864 (99.7%)	0.090
Yes	11 (0.7%)	6 (0.3%)	
Heart failure			
No	1461 (98.1%)	1858 (99.4%)	0.001
Yes	28 (1.9%)	12 (0.6%)	
Acute ischemia			
No	1489 (100.0%)	1864 (99.7%)	0.029
Yes	0 (0.0%)	6 (0.3%)	
Pulmonary embolism			
No	1464 (98.3%)	1857 (99.3%)	0.007
Yes	25 (1.7%)	13 (0.7%)	
Stroke			
No	1470 (98.7%)	1848 (98.8%)	0.794
Yes	19 (1.3%)	22 (1.2%)	
Malnutrition			
No	1406 (94.4%)	1780 (95.2%)	0.321
Yes	83 (5.6%)	90 (4.8%)	
Sepsis			
No	1429 (96.0%)	1791 (95.8%)	0.778
Yes	60 (4.0%)	79 (4.2%)	
30-day mortality			
No	1470 (98.7%)	1849 (98.9%)	0.685
Yes	19 (1.3%)	21 (1.1%)	
Reoperation			
No	1486 (99.8%)	1865 (99.7%)	0.697
Yes	3 (0.2%)	5 (0.3%)	
Prolonged length of stay			
<14 days	1350 (90.6%)	1703 (90.7%)	0.939
≥14 days	140 (9.4%)	175 (9.3%)	

## Data Availability

The use of the data from the French hospital database by our department was approved by the National Committee for data protection. We are not allowed to transmit these data. PMSI data are available for researchers who meet the criteria for access to these French confidential data (this access is submitted for the approval of the National Committee for data protection) from the national agency for the management of hospitalization (ATIH—Agence technique de l’information sur l’hospitalisation).

## Supplementary Materials

The following supporting information can be downloaded at: https://www.mdpi.com/article/10.3390/cancers16244221/s1, Table S1: Estimating the learning curve for robot-assisted lung cancer surgery; Table S2: Classification of Complications according to Clavien–Dindo; Table S3: Logistic model regression for Clavien–Dindo > II to estimate predicted risk of failure (Clavien–Dindo classification > II) in patients operated by Robot-Assisted Thoracic Surgery; Figure S1: Spline analysis using mixed-effects logistic regression for 28 hospitals that performed at least 25 robot-assisted thoracic surgeries from 2019 to 2022. Reference [27] is cited in the supplementary materials.
