# Supplementary material for: Evaluation of the Learning Curve Threshold in Robot-Assisted Lung Cancer Surgery: A Nationwide Population-Based Study
Source: Cancers (Basel). 2024 Dec 18;16(24):4221. doi: 10.3390/cancers16244221 (PMC11674775; doi:10.3390/cancers16244221)

Supplementary Table 1. Estimating the learning curve for robot-assisted lung cancer surgery

Author	Year	Effectifs	outcome	method	Threshold
SEM. Andersson et al (1)	2021	75	Duration of the operation	CUSUM	45
G Song et al. (2)	2019	208	Operative time	CUSUM	32
J J A R Baldonado et al. (3)	2018	272	Perte sanguine intra opératoire	Regression lineaire	120
C Le Gac et al (4)	2019	102	Operative time	CUSUM	27
Y Zhang et al (5)	2019	104	Operative time	Risk-adjusted CUSUM	40
M Z Yang et al (6)	2021	100	Operative time	Risk-adjusted CUSUM	56

1. SEM. Andersson et al Learning curve in robotic-assisted lobectomy for non-small cell lung cancer is not steep after experience in video-assisted lobectomy; single-surgeon experience using cumulative sum analysis. Cancer Treatment and Research Communications 2021 ; 27 : 100362 https://doi.org/10.1016/j.ctarc.2021.100362
2. G Song et al. Learning curve for robot-assisted lobectomy of lung cancer. *J Thorac Dis* 2019;11(6):2431-2437 | http://dx.doi.org/10.21037/jtd.2019.05.71
3. J J A R Baldonado et al. Credentialing for robotic lobectomy: what is the learning curve? A retrospective analysis of 272 consecutive cases by a single surgeon Journal of Robotic Surgery (2019) 13:663–669 https://doi.org/10.1007/s11701-018-00902-1
4. C Le Gac et al Medico-economic impact of robot-assisted lung segmentectomy: what is the cost of the learning curve? Interactive CardioVascular and Thoracic Surgery 2020 ; 30 : 255–262
5. Y Zhang et al Robotic Anatomical Segmentectomy: An Analysis of the Learning Curve Ann Thorac Surg 2019;107:1515–22
6. M Z Yang et al Learning curve of robotic portal lobectomy for pulmonary neoplasms: A prospective observational study Thorac Cancer. 2021;12:1431–1440.

Supplementary Table 2. Classification of Complications according to Clavien-Dindo

Grade	Definition
Grade I	Any complication without need for pharmacologic treatment or other intervention
Grade II	Any complication that requires pharmacologic treatment or minor intervention only.
Grade III	Any complication that requires surgical, radiologic, endoscopic intervention, or multitherapy.
Grade IIIa	Intervention does not require general anesthesia.
Grade IIIb	Intervention requires general anesthesia.
Grade IV	Any complication requiring intensive care unit management and life support
Grade IVa	Single organ dysfunction
Grade IVb	Multiorgan dysfunction
Grade V	Any complication leading to the death of the patient.

Supplementary Table 3. Logistic model regression for Clavien-Dindo ≥ II to estimate predicted risk of failure (clavien-dindo classification ≥ II) in patients operated by Robot-Assisted Thoracic Surgery

	Coefficient	P-value	95% Confidence Interval	
Age (years)	.0068	0.2	-.005	.018
Female	-.01	0.9	-.23	.208
Pulmonary disease	1.32	0.000	1.09	1.54
Heart disease	.64	0.000	.36	.92
Peripheral vascular	.30	0.094	-.05	.65
Neurological disease	1.12	0.000	.68	1.55
Cirrhosis	-.072	0.9	-1.11	.97
Renal disease	-.15	0.6	-.72	.41
Metabolic disease	.16	0.3	-.14	.46
Infectious disease	.275	0.8	-2.03	2.56
Hematologic disease	.55	0.11	-.13	1.23
Other disease	.055	0.7	-.23	.34
Other treatment	-.084	0.6	-.41	.245
Charlson score				
1	-.083	0.65	-.44	.28
2	-.44	0.03	-.84	-.04
>=3	-.20	0.26	-.55	.13
Pulmonary resection				
Lobectomy	5.16	0.000	4.27	6.05
Year				
2020	-.156	0.54	-.66	.35
2021	-.12	0.63	-.60	.36
2022	-.15	0.54	-.63	.36
Intercept	-11.46	0.000	-13	-10

Supplementary Figure 1. Spline analysis using mixed-effects logistic regression for 28 hospitals that performed at least 25 robot-assisted thoracic surgeries from 2019 to 2022.

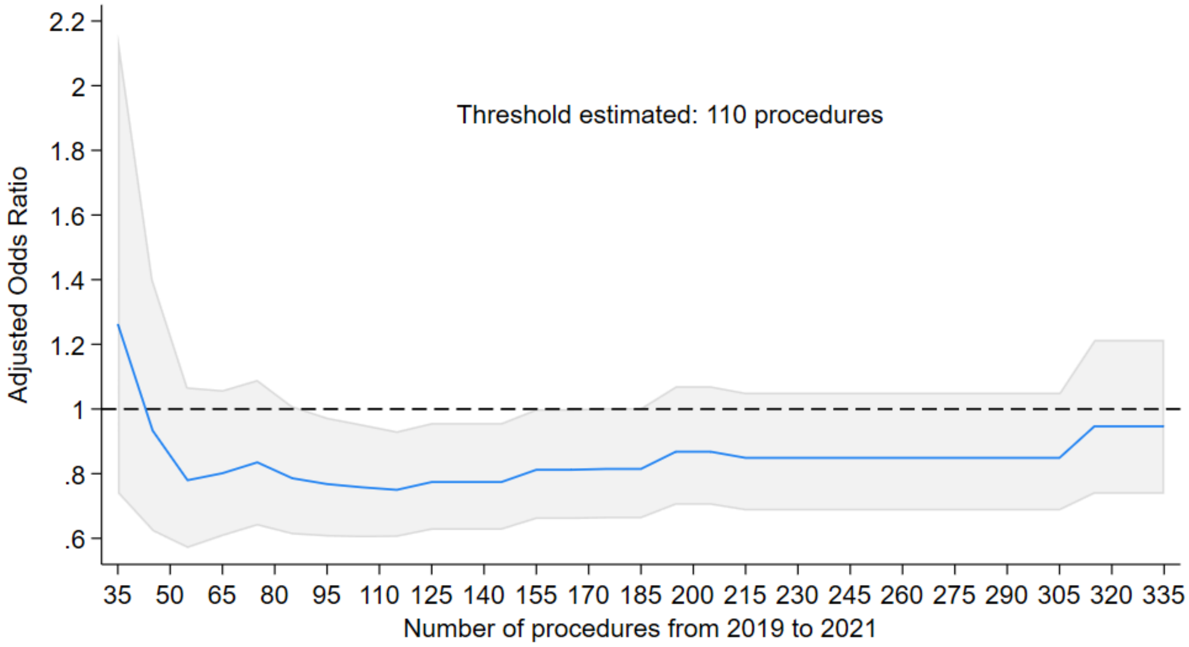

Supplement: Supplementary file 1 [file cancers-16-04221-s001.zip › cancers-3326183-supplementary.pdf]
